# Low use of routine medical care among African Americans with high CKD risk: the Jackson Heart Study

**DOI:** 10.1186/s12882-018-1190-0

**Published:** 2019-01-10

**Authors:** Clarissa J. Diamantidis, Clemontina A. Davenport, Joseph Lunyera, Nrupen Bhavsar, Julia Scialla, Rasheeda Hall, Crystal Tyson, Mario Sims, Tara Strigo, Neil R. Powe, L. Ebony Boulware

**Affiliations:** 1 Division of General Internal Medicine, Duke University School of Medicine, Durham, NC USA; 2 Division of Nephrology, Duke University School of Medicine, 411 W. Chapel Hill St, Suite 500, Durham, NC 27701 USA; 3 Department of Biostatistics and Bioinformatics, Duke University School of Medicine, Durham, NC USA; 4 Duke Clinical Research Institute, Duke University, Durham, NC USA; 5 Jackson Heart Study, University of Mississippi School of Medicine, Jackson, MS USA; 6 University of California at San Francisco School of Medicine, San Francisco, CA USA

**Keywords:** Chronic kidney disease, Trust, Routine care

## Abstract

**Background:**

Use of routine medical care (RMC) is advocated to address ethnic/racial disparities in chronic kidney disease (CKD) risks, but use is less frequent among African Americans. Factors associated with low RMC use among African Americans at risk of renal outcomes have not been well studied.

**Methods:**

We examined sociodemographic, comorbidity, healthcare access, and psychosocial (discrimination, anger, stress, trust) factors associated with low RMC use in a cross-sectional study. Low RMC use was defined as lack of a physical exam within one year among participants with CKD (estimated glomerular filtration rate < 60 mL/min/1.73m^2^ or urine albumin-to-creatinine ratio > 30 mg/g) or CKD risk factors (diabetes or hypertension). We used multivariable logistic regression to estimate the odds of low RMC use at baseline (2000–2004) for several risk factors.

**Results:**

Among 3191 participants with CKD, diabetes, or hypertension, 2024 (63.4%) were ≥ 55 years of age, and 700 (21.9%) reported low RMC use. After multivariable adjustment, age < 55 years (OR 1.61 95% CI 1.31–1.98), male sex (OR 1.71; 1.41–2.07), <high school diploma (OR 1.31; 1.07–1.62), absence of hypertension (OR 1.74; 1.27–2.39) or diabetes (OR 1.34; 1.09–1.65), and tobacco use (OR 1.43; 1.18–1.72) were associated with low RMC use. Low trust in providers (OR 2.16; 1.42–3.27), high stress (OR 1.41; 1.09–1.82), high daily discrimination (OR 1.30; 1.01–1.67) and low burden of lifetime discrimination (OR 1.52; 1.18–1.94), were also associated with low RMC use.

**Conclusions:**

High-risk African Americans who were younger, male, less-educated, and with low trust in providers were more likely to report low RMC use. Efforts to improve RMC use by targeting these populations could mitigate African Americans’ disparities in CKD risks.

**Electronic supplementary material:**

The online version of this article (10.1186/s12882-018-1190-0) contains supplementary material, which is available to authorized users.

## Background

African Americans, especially those with diabetes, hypertension, or a family history of chronic kidney disease (CKD), have two- to four-fold greater incidence of end stage renal disease (ESRD) or death when compared to their non-minority counterparts with similar CKD risk factors [1–3]. Poor access to health care, more commonly reported among ethnic/racial minorities than non-minorities, is thought to partially contribute to African Americans’ excess CKD-related health risks [4, 5]. Receipt of routine medical care (RMC) facilitates receipt of important preventive care services and health education, [6] with lack of RMC contributing to ethnic/racial disparities in these services [7, 8].

While greater use of RMC is widely advocated as a potential solution to address disparities in CKD, African Americans at risk of CKD have been shown to use RMC less frequently than non-African Americans [5]. However, reasons for low use of RMC among African Americans with CKD risk factors are not well-understood. In studies of diverse populations, individuals with CKD risk factors such diabetes or hypertension have been known to have low perceived susceptibility to CKD [9–11], and those with established kidney dysfunction have low rates of CKD awareness [12, 13]. These factors may contribute to limited engagement with the health care process. Little is known about how other sociodemographic factors, comorbidity, healthcare access (e.g. health insurance and type of coverage), or psychosocial factors (e.g. anger or stress) contribute to suboptimal RMC use among African Americans at risk of CKD. Improved understanding of additional factors associated with low use of RMC could inform efforts to eliminate disparities in CKD outcomes among African Americans.

We assessed low use of RMC among African Americans enrolled in the Jackson Heart Study (JHS) who were at risk of CKD incidence or progression. The JHS collects a unique battery of psychosocial questionnaires including assessments of stress, discrimination, anger and trust. Using this rich data we assess participants’ demographic, medical, socioeconomic and psychosocial characteristics associated with use of RMC thereby providing a more comprehensive evaluation of factors affecting RMC in African Americans with or at risk for CKD than currently available in the literature. Given the high burden of CKD in this group, understanding barriers to care will be critical to implementation of preventive interventions.

## Methods

### Study population

The JHS is a prospective cohort study of cardiovascular disease (CVD) in African American residents living in the tri-county area (Hinds, Madison, and Rankin) of the metropolitan statistical area of Jackson, Mississippi. Detailed study procedures and recruitment are described elsewhere [14, 15]. Briefly, African Americans living in Jackson, Mississippi – including participants from the Atherosclerosis Risk in Communities (ARIC) and their family members, were recruited and examined during the JHS baseline visit (2000–2004), comprising a final baseline cohort of 5306 African Americans 21–94 years of age. Participants completed questionnaires which captured information on socio-demographics, comorbidity, healthcare utilization, and psychosocial factors including trust in their medical care and, moods such as anger, perceived stress, and perceived discrimination. Participants also underwent standard physical examinations (including blood pressure [BP] measurement) and laboratory studies (including measures of kidney function and glycemic control). Each participant provided written informed consent, and the institutional review boards at the University of Mississippi Medical Center, Jackson State University, and Tougaloo College approved the JHS study protocol.

### Assessment of comorbidities

We identified individuals with CKD, hypertension or diabetes in the JHS cohort during the baseline visit. Using the JNC 7 criteria for detection of hypertension [16], we included individuals with measured BP > 140/90 mmHg or with self-reported use of BP lowering medication. We defined diabetes as fasting glucose ≥126 mg/dL, hemoglobin A1C ≥6.5%, or use of diabetes medications within 2 weeks prior to the clinic visit [17]. We defined CKD as the presence of an estimated glomerular filtration rate (eGFR) < 60 mL/min/1.73m^2^ using the CKD-EPI formula [18] or a urine albumin-to-creatinine ratio (UACR) > 30 mg/g on spot collection or 24 h urine collection if a spot urine collection was unavailable. We included only individuals with complete demographic, comorbidity, and psychosocial data in the analysis.

### Assessment of psychosocial factors

We included several baseline psychosocial factors in the present analysis: trust in medical care, perceived discrimination, anger, and stress. Among participants who self-reported access to health care, we ascertained their trust in medical care using the question: “Thinking about the place you usually go for help with your medical problems, in general, how much do you trust them to take good care of you? Do you trust them very much, somewhat, not very much, or not at all?” We considered participants responding “very much” or “somewhat” to have high trust in medical care, and those responding “not very much” or “not at all” to have low trust in medical care.

The multidimensional Jackson Heart Study Discrimination Instrument (JHSDIS) – developed specifically for use in the JHS cohort – assessed perceived social discrimination [19]. The JHSDIS evaluated three dimensions of perceived discrimination: daily discrimination, lifetime discrimination, and burden of lifetime discrimination. Daily discrimination consisted of responses to 9 statements each prefaced with the question: “How often on a day-to-day basis do you have the following experiences?” Statements captured treatment with less courtesy, or less respect; receipt of poor service at a restaurant; and several examples of profiling with regard to factors such as intelligence, hostility/violence, honesty, and others. Participants rated the frequency of these experiences on a scale from 0 (no experiences) to 6 (several times a day) and responses were compiled into a summary score.

To assess lifetime discrimination, participants were asked to answer yes or no to questions about unfair treatments over their lifetime across 9 domains capturing aspects of daily living such as school, job search, workplace, buying a house, accessing resources/services, including medical care, and use of public spaces. The count of the domains (range 0–9) was the lifetime social discrimination score. The burden of lifetime social discrimination was based on a summed score of the points in response to 3 questions: “when you have had experiences like these over your lifetime, would you say they have been very stressful [3 points], moderately stressful [1.5 points], or not stressful [0 points]?”, “overall, how much has discrimination interfered with you having a full and productive life? Would you say a lot [3 points], some, a little, or not at all [0 points]?”, and “overall, how much harder has your life been because of discrimination? Would you say a lot [3 points], some [2 points], a little [1 points], or not at all [0 points]?” We categorized overall scores for daily, lifetime, and burden of lifetime discrimination into tertiles.

Anger was measured using the Spielberger trait anger scale, a 16-item scale that assesses anger-in (8 items) and anger-out (8 items) [20]. Using a Likert scale (“almost never”, “sometimes”, “often”, and “almost always”), participants rated their anger reactions such as “I do things like slam doors,” or “I am secretly quite critical of others.” We summed responses within each subscale, and averaged the two summed scores as the overall anger score, which was then categorized into tertiles.

Participants’ responses to the Global Perceived Stress Scale – an 8-item measure of perceived chronic stress created for use in the JHS – assessed psychosocial stress [21]. Participants rated the severity of stress experienced over the 12 months prior to the baseline exam in 8 areas from 0 [not stressful] to 3 [very stressful]: employment, relationships, neighborhood, caring for others, legal problems, medical problems, racism and discrimination, and meeting basic needs [22]. We summed responses and categorized the composite score into tertiles.

### Assessment of healthcare access and utilization

Participants self-reported their health insurance status and type of coverage. Participants also indicated difficulty obtaining healthcare services in response to the question “overall, how hard has it been for you to get health services you have needed? Would you say it has been very hard, fairly hard, not too hard, or not hard at all?” We considered those who responded “very hard” or “fairly hard” as having difficulty obtaining services. Participants rated their satisfaction with care based on their response to the question “overall, how satisfied are you with your regular (or most recent) doctor or health professional? Would you say you are very satisfied, somewhat satisfied, somewhat dissatisfied, very dissatisfied, or not sure?” We considered those who responded with “very satisfied” or “somewhat satisfied” to be satisfied with their care.

### Outcome assessment: Low use of routine medical care

We defined participants’ low use of RMC based on their responses to the question: “when was the last time you went to a doctor or other health professional for a routine physical exam or general check-up; that is when you were not sick or pregnant?” Possible responses were “within the past year”, “at least 1 year but less than 2 years ago”, “at least 2 years but less than 4 years ago”, “5 or more years ago”, or “never.” We considered participants who reported no receipt of a routine physical exam “within the past year” to have low use of RMC.

### Statistics

In descriptive analyses, we summarized characteristics of study participants and compared these characteristics between those who reported low use vs. use of RMC using Chi-square tests. Because these analyses were descriptive, no adjustment for multiple testing was made. Using a multivariable logistic regression model, we estimated the odds of low RMC use by sociodemographic, comorbidity, healthcare access, and psychosocial factors. Multivariable models were adjusted for age, sex, education, income, insurance, diabetes, hypertension, CVD history, smoking status, BMI, and CKD status. In a post hoc analysis exploring the impact of age on low use of RMC, we stratified the regression model by age (dichotomized at the cohort median: < 55 vs ≥55 years). All tests were two-sided at significance level α < 0.05. All analyses were performed using SAS 9.4 (SAS Institute, Cary, NC) and R 3.3.0 (R Core Team, Vienna, Austria).

## Results

### Study participants, sociodemographics, and comorbidity

**Fig. 1:**
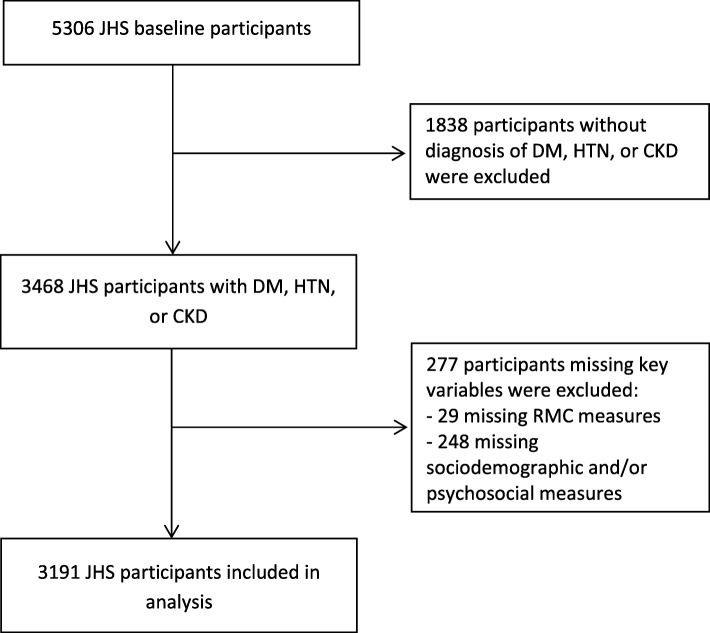
Derivation of analytic cohort

Of 5306 total JHS participants enrolled at baseline, 3468 (65.4%) had CKD, diabetes, or hypertension. Of these, we included 3191 participants who had complete data available for assessment of RMC use, access to care, and psychosocial factors (Fig. 1). Compared with available data on excluded participants, participants who met inclusion criteria were older, with less education, lower income, and more prevalent CVD, and were more likely to be insured. Included participants reported lower stress, daily discrimination, and burden of lifetime discrimination than those excluded from the analysis (Additional file 1: Table S1).

Among those included in the analysis, 700 (21.9%) reported low use of RMC. Participants reporting low use of RMC were more likely to be younger (age < 55 years: 48.6% vs. 33.2%), male (47.6% vs. 32.5%), or former/current smokers (42.6% vs. 32.8%) than those reporting use of RMC. Compared to those with RMC use, participants with low RMC use were less likely overall to have hypertension (88.3% vs. 92.9%) or diabetes (29.1% vs. 34.7%), but more likely to have uncontrolled hypertension (60.7% vs. 47.2%). Of 591 (18.5%) participants with CKD, those with low use of RMC were slightly less likely to have later stages of CKD than those with use of RMC (CKD stage 4 or 5: 3.9% vs. 7.6%). Few participants with CKD reported CKD awareness (13.7%), and there was no difference in CKD awareness between participants with or without use of RMC (14.3% vs. 11.7%) (Table 1).

**Table 1:** Baseline characteristics of participants, stratified by use of routine medical care

Characteristic	All participants *N* = 3191				Age < 55 years *N* = 1167 (36.6%)			Age ≥ 55 years *N* = 2024 (63.4%)		
	Overall	RMC use *N* = 2491 (78.1%)	Low RMC use *N* = 700 (21.9%)	*P* Value	RMC use *N* = 827 (33.2%)	Low RMC use *N* = 340 (48.6%)	*P* Value	RMC use *N* = 1664 (66.8%)	Low RMC use *N* = 360 (51.4%)	*P* Value
Sociodemographic										
Age, years, mean ± SD	58.9 (11.39)	59.85 (11.19)	55.55 (11.47)	< 0.01	47 (5.93)	45.67 (6.31)	< 0.01	66.23 (6.86)	64.89 (6.21)	< 0.01
Sex				< 0.01			< 0.01			< 0.01
Female	2049 (64.21)	1682 (67.52)	367 (52.43)		542 (65.54)	158 (46.47)		1140 (68.51)	209 (58.06)	
Male	1142 (35.79)	809 (32.48)	333 (47.57)		285 (34.46)	182 (53.53)		524 (31.49)	151 (41.94)	
Education				0.09			< 0.01			< 0.01
≤ High school diploma	1389 (43.53)	1064 (42.71)	325 (46.43)		184 (22.25)	103 (30.29)		880 (52.88)	222 (61.67)	
> High school diploma	1802 (56.47)	1427 (57.29)	375 (53.57)		643 (77.75)	237 (69.71)		784 (47.12)	138 (38.33)	
Income class category^a^				0.10			0.37			0.05
Lower/Lower-middle	1139 (35.69)	876 (35.17)	263 (37.57)		218 (26.36)	103 (30.29)		658 (39.54)	160 (44.44)	
Upper-middle/Upper	1548 (48.51)	1204 (48.33)	344 (49.14)		472 (57.07)	186 (54.71)		732 (43.99)	158 (43.89)	
Missing	504 (15.79)	411 (16.5)	93 (13.29)		137 (16.57)	51 (15)		274 (16.47)	42 (11.67)	
Comorbidities & Behaviors										
BMI, kg/m^2^				0.65			0.47			0.99
< 30	1316 (41.24)	1033 (41.47)	283 (40.43)		260 (31.44)	115 (33.82)		773 (46.45)	168 (46.67)	
≥ 30	1875 (58.76)	1458 (58.53)	417 (59.57)		567 (68.56)	225 (66.18)		891 (53.55)	192 (53.33)	
Tobacco use				< 0.01			< 0.01			< 0.01
Never	2076 (65.06)	1674 (67.2)	402 (57.43)		604 (73.04)	209 (61.47)		1070 (64.3)	193 (53.61)	
Former/current	1115 (34.94)	817 (32.8)	298 (42.57)		223 (26.96)	131 (38.53)		594 (35.7)	167 (46.39)	
Hypertension				< 0.01			0.01			0.03
No	259 (8.12)	177 (7.11)	82 (11.71)		72 (8.71)	47 (13.82)		105 (6.31)	35 (9.72)	
Yes	2932 (91.88)	2314 (92.89)	618 (88.29)		755 (91.29)	293 (86.18)		1559 (93.69)	325 (90.28)	
Hypertension control^b^				< 0.01			0			0
Controlled	1456 (49.66)	1215 (52.51)	241 (39)		392 (51.92)	103 (35.15)		823 (52.79)	138 (42.46)	
Uncontrolled	1467 (50.03)	1092 (47.19)	375 (60.68)		363 (48.08)	188 (64.16)		729 (46.76)	187 (57.54)	
Diabetes				0.01			0.37			0.09
No	2122 (66.5)	1626 (65.27)	496 (70.86)		600 (72.55)	256 (75.29)		1026 (61.66)	240 (66.67)	
Yes	1069 (33.5)	865 (34.73)	204 (29.14)		227 (27.45)	84 (24.71)		638 (38.34)	120 (33.33)	
Diabetes control^c^				0.66			0.4			0.8
Controlled	475 (44.43)	381 (44.05)	94 (46.08)		83 (36.56)	37 (44.05)		298 (46.71)	57 (47.5)	
Uncontrolled	556 (52.01)	453 (52.37)	103 (50.49)		133 (58.59)	46 (54.76)		320 (50.16)	57 (47.5)	
CVD history				0.17			0.09			0.5
No	2741 (85.9)	2128 (85.43)	613 (87.57)		764 (92.38)	324 (95.29)		1364 (81.97)	289 (80.28)	
Yes	450 (14.1)	363 (14.57)	87 (12.43)		63 (7.62)	16 (4.71)		300 (18.03)	71 (19.72)	
CKD				0.73			0.33			0.13
No	1500 (47.01)	1178 (47.29)	322 (46)		467 (56.47)	183 (53.82)		711 (42.73)	139 (38.61)	
Yes	591 (18.52)	463 (18.59)	128 (18.29)		113 (13.66)	58 (17.06)		350 (21.03)	70 (19.44)	
CKD stage ^d^				0.01			0.35			0.46
1 or 2	297 (50.25)	217 (46.87)	80 (62.5)		87 (76.99)	50 (86.21)		130 (37.14)	30 (42.86)	
3	251 (42.47)	209 (45.14)	42 (32.81)		18 (15.93)	6 (10.34)		191 (54.57)	36 (51.43)	
4 or 5	40 (6.77)	35 (7.56)	5 (3.91)		8 (7.08)	2 (3.45)		27 (7.71)	3 (4.29)	
CKD awareness ^d,e^				0.57			0.1			0.59
No	508 (85.96)	396 (85.53)	112 (87.5)		95 (84.07)	55 (94.83)		301 (86)	57 (81.43)	
Yes	81 (13.71)	66 (14.25)	15 (11.72)		17 (15.04)	3 (5.17)		49 (14)	12 (17.14)	
Healthcare access & utilization										
Health insurance				< 0.01			< 0.01			< 0.01
No	385 (12.07)	253 (10.16)	132 (18.86)		124 (14.99)	84 (24.71)		129 (7.75)	48 (13.33)	
Yes	2806 (87.93)	2238 (89.84)	568 (81.14)		703 (85.01)	256 (75.29)		1535 (92.25)	312 (86.67)	
Insurance type ^f^										
Medicare	1128 (35.35)	953 (38.26)	175 (25)	< 0.01	64 (7.74)	9 (2.65)	< 0.01	889 (53.43)	166 (46.11)	0.01
Medicaid	479 (15.01)	393 (15.78)	86 (12.29)	0.03	62 (7.5)	17 (5)	0.16	331 (19.89)	69 (19.17)	0.81
Private	2052 (64.31)	1616 (64.87)	436 (62.29)	0.22	622 (75.21)	241 (70.88)	0.14	994 (59.74)	195 (54.17)	0.06
Usual source of care				< 0.01			< 0.01			< 0.01
No	200 (6.27)	93 (3.73)	107 (15.29)		41 (4.96)	66 (19.41)		52 (3.12)	41 (11.39)	
Yes	2980 (93.39)	2388 (95.87)	592 (84.57)		783 (94.68)	274 (80.59)		1605 (96.45)	318 (88.33)	
Obtaining care				< 0.01			< 0.01			0.02
Less difficult	2794 (87.56)	2223 (89.24)	571 (81.57)		723 (87.42)	263 (77.35)		1500 (90.14)	308 (85.56)	
More difficult	383 (12)	262 (10.52)	121 (17.29)		102 (12.33)	72 (21.18)		160 (9.62)	49 (13.61)	
Satisfaction with care				< 0.01			< 0.01			< 0.01
Less satisfied	154 (4.83)	88 (3.53)	66 (9.43)		40 (4.84)	38 (11.18)		48 (2.88)	28 (7.78)	
More satisfied	2992 (93.76)	2393 (96.07)	599 (85.57)		784 (94.8)	278 (81.76)		1609 (96.69)	321 (89.17)	

*RMC* routine medical care, *BMI* body mass index, *CVD* cardiovascular disease, *CKD* chronic kidney disease

^a^Based on family size, US Census poverty levels, and year of baseline clinic visit (2000–2004). Among participants with ^b^ hypertension, ^c^ diabetes, ^d,e^ CKD (< 100% due to *n* = 3 who could not be staged and *n* = 2 missing self-report data); ^f^health insurance categories are not mutually exclusive

### Healthcare access and utilization

Most participants had health insurance (*n* = 2806; 87.9%). Compared to participants with use of RMC, those with low RMC use were less likely to report having health insurance (81.1% vs. 89.8%) or a usual source of medical care (84.6% vs. 95.9%), but were more likely to report difficulty obtaining care (17.3% vs. 10.5%) and being less satisfied with care (9.4% vs. 3.5%; Table 1).

### Distribution of psychosocial factors

Self-reported psychosocial factors are shown in Table 2. Overall, participants reporting low use of RMC were more likely to report low (vs. high) trust in their medical care compared to those with use of RMC (7.0% vs. 2.9%), although overall trust was high. Those with low use of RMC were more commonly in the highest tertile of stress score (44.3% vs. 36.9%) and daily discrimination score (41.3% vs. 33.5%) than participants reporting RMC use. Trust remained lower among individuals with low RMC use compared with RMC use in both the < 55 years old group and ≥ 55 year old groups. However, among participants aged ≥55 years, those with low use of RMC had lower scores on the burden of lifetime discrimination measure (lowest tertile: 37.5% vs. 30.5%) compared to those with use of RMC (Table 2).

**Table 2:** Distribution of psychosocial factors among participants in Jackson Heart Study at baseline overall; stratified by use of routine medical care

Psychosocial factors	All participants *N* = 3191				Age < 55 years *N* = 1167 (36.6%)			Age ≥ 55 years *N* = 2024 (63.4%)		
	Overall	RMC use *N* = 2491 (78.1%)	Low RMC use *N* = 700 (21.9%)	*P* Value	RMC use *N* = 827 (33.2%)	Low RMC use *N* = 340 (48.6%)	*P* Value	RMC use *N* = 1664 (66.8%)	Low RMC use *N* = 360 (51.4%)	*P* Value
Trust in care^a^				< 0.01			0			0.03
Low	111 (3.72)	70 (2.93)	41 (6.95)		28 (3.56)	25 (9.12)		42 (2.62)	16 (5.06)	
High	2870 (96.28)	2321 (97.07)	549 (93.05)		758 (96.44)	249 (90.88)		1563 (97.38)	300 (94.94)	
Stress tertile				< 0.01			0.44			0.31
Lowest	859 (26.92)	695 (27.9)	164 (23.43)		107 (12.94)	45 (13.24)		588 (35.34)	119 (33.06)	
Middle	1102 (34.53)	876 (35.17)	226 (32.29)		287 (34.7)	105 (30.88)		589 (35.4)	121 (33.61)	
Highest	1230 (38.55)	920 (36.93)	310 (44.29)		433 (52.36)	190 (55.88)		487 (29.27)	120 (33.33)	
Anger tertile				0.11			0.19			0.17
Lowest	626 (19.62)	505 (20.27)	121 (17.29)		135 (16.32)	46 (13.53)		370 (22.24)	75 (20.83)	
Middle	615 (19.27)	485 (19.47)	130 (18.57)		181 (21.89)	76 (22.35)		304 (18.27)	54 (15)	
Highest	768 (24.07)	604 (24.25)	164 (23.43)		258 (31.2)	94 (27.65)		346 (20.79)	70 (19.44)	
Discrimination tertiles										
Daily				< 0.01			0.42			0.06
Lowest	988 (30.96)	793 (31.83)	195 (27.86)		166 (20.07)	63 (18.53)		627 (37.68)	132 (36.67)	
Middle	1080 (33.85)	864 (34.68)	216 (30.86)		285 (34.46)	108 (31.76)		579 (34.8)	108 (30)	
Highest	1123 (35.19)	834 (33.48)	289 (41.29)		376 (45.47)	169 (49.71)		458 (27.52)	120 (33.33)	
Lifetime				0.32			0.57			0.13
Lowest	894 (28.02)	704 (28.26)	190 (27.14)		164 (19.83)	66 (19.41)		540 (32.45)	124 (34.44)	
Middle	1104 (34.6)	845 (33.92)	259 (37)		273 (33.01)	123 (36.18)		572 (34.38)	136 (37.78)	
Highest	1193 (37.39)	942 (37.82)	251 (35.86)		390 (47.16)	151 (44.41)		552 (33.17)	100 (27.78)	
Burden of lifetime				0.09			0.49			0.04
Lowest	963 (30.18)	731 (29.35)	232 (33.14)		223 (26.96)	97 (28.53)		508 (30.53)	135 (37.5)	
Middle	1093 (34.25)	853 (34.24)	240 (34.29)		301 (36.4)	131 (38.53)		552 (33.17)	109 (30.28)	
Highest	1135 (35.57)	907 (36.41)	228 (32.57)		303 (36.64)	112 (32.94)		604 (36.3)	116 (32.22)	

*RMC* routine medical care

^a^Among participants reporting a usual source of care and non-missing response to question regarding trust (*n* = 2981)

### Factors independently associated with low use of routine medical care

In a multivariable logistic regression model adjusted for sociodemographics, comorbidities, and psychosocial factors, age < 55 years (OR 1.61; 95% CI 1.31–1.98), male sex (OR 1.71; 1.41–2.07), < high school diploma (OR 1.31; 1.07–1.62), and lack of health insurance (OR 1.52; 1.18–1.96) were associated with greater odds of low use of RMC overall (Table 3). Among participants ≥55 years, male sex and lack of health insurance were associated with greater odds of low RMC use (OR 1.56; 1.20–2.04, OR 1.69; 1.16–2.46, respectively), while male sex was significantly associated only in the < 55 years age group (OR 1.90; 1.43–2.54).

**Table 3:** Factors associated with low use of routine medical care (RMC); stratified by age

	Odds Ratio (95% confidence interval)		
	All participants	Age < 55 years	Age ≥ 55 years
No. of events/N	700/3191	340/1167	360/2024
Sociodemographic characteristics			
Age			
< 55 years	1.61 (1.31, 1.98)	NA	NA
≥ 55 years	Reference	NA	NA
Gender			
Male	1.71 (1.41, 2.07)	1.90 (1.43, 2.54)	1.56 (1.20, 2.04)
Female	Reference	Reference	Reference
Education			
< high school diploma	1.31 (1.07, 1.62)	1.37 (0.98, 1.91)	1.30 (0.99, 1.70)
≥ high school diploma	Reference	Reference	Reference
Income category			
Poor: lower-middle	0.97 (0.78, 1.20)	0.95 (0.68, 1.34)	0.96 (0.72, 1.27)
Affluent: upper-middle	Reference	Reference	Reference
Health insurance			
No	1.52 (1.18, 1.96)	1.40 (0.98, 2.01)	1.69 (1.16, 2.46)
Yes	Reference	Reference	Reference
Comorbidities & Behaviors			
Body mass index, Kg/m^2^			
< 30	Reference	Reference	Reference
≥ 30	1.17 (0.97, 1.42)	1.08 (0.80, 1.45)	1.25 (0.97, 1.61)
Tobacco use			
Never	Reference	Reference	Reference
Former/current	1.43 (1.18, 1.72)	1.50 (1.12, 2.03)	1.44 (1.12, 1.84)
Hypertension			
No	1.74 (1.27, 2.39)	1.71 (1.08, 2.71)	1.79 (1.15, 2.79)
Yes	Reference	Reference	Reference
Diabetes			
No	1.34 (1.09, 1.65)	1.24 (0.88, 1.73)	1.41 (1.08, 1.84)
Yes	Reference	Reference	Reference
Cardiovascular disease			
No	1.12 (0.85, 1.47)	1.89 (1.04, 3.42)	0.93 (0.68, 1.27)
Yes	Reference	Reference	Reference
Chronic kidney disease			
No	0.96 (0.75, 1.24)	0.81 (0.54, 1.21)	1.06 (0.76, 1.49)
Yes	Reference	Reference	Reference
Psychosocial factors			
Trust ^a^			
Low	2.16 (1.42, 3.27)	2.32 (1.29, 4.16)	2.07 (1.13, 3.80)
High	Reference	Reference	Reference
Stress tertile			
Lowest	Reference	Reference	Reference
Middle	1.16 (.91, 1.48)	1.03 (0.66, 1.62)	1.18 (0.88, 1.58)
Highest	1.41 (1.09, 1.82)	1.29 (0.82, 2.01)	1.41 (1.02, 1.94)
Anger tertile			
Lowest	Reference	Reference	Reference
Middle	0.95 (0.71, 1.28)	1.15 (0.73, 1.81)	0.83 (0.55, 1.23)
Highest	0.87 (0.65, 1.15)	0.88 (0.56, 1.37)	0.89 (0.61, 1.29)
Daily discrimination tertile			
Lowest	Reference	Reference	Reference
Middle	1.05 (0.83, 1.34)	1.10 (0.73, 1.66)	1.03 (0.76, 1.40)
Highest	1.30 (1.01, 1.67)	1.15 (0.76, 1.75)	1.46 (1.05, 2.03)
Lifetime discrimination tertile			
Lowest	Reference	Reference	Reference
Middle	1.10 (0.86, 1.40)	1.16 (0.76, 1.76)	1.09 (0.81, 1.48)
Highest	0.89 (0.68, 1.17)	1.08 (0.70, 1.68)	0.76 (0.53, 1.08)
Burden of discrimination tertile			
Lowest	Reference	Reference	Reference
Middle	0.82 (0.65, 1.03)	0.93 (0.65, 1.34)	0.74 (0.54, 1.00)
Highest	0.66 (0.51, 0.85)	0.70 (0.47, 1.05)	0.64 (0.46, 0.89)

All models were adjusted for the covariates listed above

^a^Among participants reporting a usual source of care and non-missing response to question regarding trust (*n* = 2981)

Among comorbidities and behaviors, tobacco use was associated with higher odds of low RMC use overall (OR 1.43; 1.18–1.72) and across age groups. Absence of comorbidities such as hypertension (OR 1.74; 1.27–2.39) or diabetes (OR 1.34; 1.09–1.65) was associated with greater odds of low use of RMC; the latter was driven by the ≥55 years age group. Participants < 55 years of age without CVD had greater odds of low RMC use than participants with CVD (OR 1.89; 1.04–3.42).

Participants reporting low trust had more than twice the odds of reporting low use of RMC compared to those with high trust (OR 2.16; 1.42–3.27) overall and across both age groups. Trust was the only psychosocial factor associated with low RMC use among participants < 55 years of age. Participants in the highest (vs. lowest) tertile of stress and daily discrimination also had higher odds of low use of RMC (OR 1.41; 1.02, 1.94, OR 1.46; 1.05–2.03, respectively), whereas participants in the highest (vs. lowest) tertile of burden of lifetime discrimination had lower odds of reporting low use of RMC (OR0.64; 0.46–0.89). After stratification by age, these findings were only evident among participants ≥55 years of age.

## Discussion

In this large cohort of African Americans with CKD or at increased risk of CKD, younger age, male sex, low educational attainment, tobacco use, lack of health insurance, and low comorbidity were associated with low use of RMC. Psychosocial factors (e.g., low trust, high stress, high daily and low burden of lifetime discrimination) were also associated with low use of RMC among this high risk group. These results suggest that interventions focused on increasing trust and reducing stressors in non-health related activities could encourage more preventive care for African Americans and promote wellness.

To our knowledge, this is the first study to simultaneously examine a number of factors associated with infrequent use of RMC among African Americans at high risk of poor CKD outcomes. The extent to which patients seek health care may directly affect their opportunities to have CKD detected and their CKD risk factors controlled, and their opportunities for education regarding the long-term health risks associated with CKD, particularly if that care is received episodically. While use of routine care creates opportunities for health education, preventive health services, disease management, and shared decision-making, these opportunities are less frequently encountered by African Americans than by whites [23, 24]. The reason for this difference is likely multifactorial. For instance, racial residential segregation may impact disparities in routine care, [25] as African Americans are more segregated from optimal healthcare facilities and information. This access to healthcare services directly relates to use of routine care [26–28]. In African American populations, this use of routine care has been linked to an identified usual source of care [7, 29], yet ethnic and racial minorities are routinely less likely to have an identified usual source of care than their white counterparts [30]. As a result, individuals without a usual source of care are more likely to utilize the emergency department for their healthcare needs than individuals with access to routine care [31], resulting in an expensive and inefficient method of obtaining health services.

Despite these findings, differences in healthcare access alone may not fully explain disparities in routine care use between African Americans and whites. Psychosocial factors and cultural norms may also influence use of routine care [29, 32, 33]. Our findings are consistent with prior literature suggesting low trust as a major barrier to health care engagement by African Americans [34]. Low trust in medical care among ethnic and racial minorities has been associated with lower medication adherence, reduced rates of preventive health services, worsened blood pressure control, and varying degrees of shared-decision making [35–40]. Factors contributing to low trust include patient perceptions of provider greed, discrimination, and the potential for medical experimentation based on the historical medical mistreatment of African Americans [41, 42]. Conversely, provider race concordance has been associated with higher levels of trust and patient engagement, [43–46] which may be due to a more positive physician affect and higher degree of person-centered communication noted more frequently in racially concordant office visits [47, 48]. It remains unclear how patterns of patient-provider interactions, for example, the degree of continuity of care by providers contribute to feelings of low trust among JHS participants. Further, we found incongruence between perceived discrimination and use of RMC, with high daily discrimination, yet low burden of lifetime discrimination to be associated with low RMC use. The latter finding contradicts our hypothesis that perceived discrimination would be associated with low use of RMC, but aligns with other JHS studies showing higher degrees of perceived burden of lifetime discrimination to be associated with more favorable outcomes (e.g. lower risk of left ventricular hypertrophy) [49]. This is hypothesized to be related to the aggregation of components in the burden of lifetime discrimination assessment, or potentially an age-related phenomenon. Evaluation of the individual questions in the burden assessment may produce different results.

Our findings suggest patterns of healthcare utilization differ between younger and older African Americans. Younger JHS participants were less engaged in RMC than older participants, aligning with prior studies showing young adults in the general population have less use of ambulatory medical care, [50, 51] and rate their health as a lower priority than adolescents or older adults [52]. Prior work has shown young adults with hypertension to be less frequently diagnosed by a provider than older adults with similar blood pressures, [53] receive lifestyle education more inconsistently, [54] and have high rates of hypertension unawareness [55]. Further, young adults with established CKD are at high risk of complications related to poor self-management skills and non-adherence, especially during transitions in care, [56] and are particularly vulnerable to poor CKD outcomes. While several studies have demonstrated improved survival on dialysis among older African Americans compared to whites, [57–60] young adult African Americans aged 18–30 have been shown to suffer a two-fold increase risk of death compared to age-matched whites and older African Americans [61, 62]. Tailored interventions promoting early engagement in RMC among young African Americans may mitigate CKD risks when the potential long-term impact on CKD outcomes is greatest.

Our study has limitations that should be noted. First, JHS participants reside in a single southeastern US metropolitan area with a high prevalence of CKD risk factors, which limits the generalizability of the study findings to African Americans in other areas of the US and to individuals residing outside of the US. Further, we did not have detailed information on specific characteristics that may influence RMC such as past personal or familiar medical experiences or provider race concordance, nor were we able to identify the directionality of our findings given the cross-sectional nature of our study. For example, we cannot determine if younger individuals were more likely to be diagnosed with CVD because of engagement in RMC or whether they were more engaged in care because of pre-existing health problems. Finally, to ensure sufficient power for outcome assessment, we chose to stratify age at the JHS cohort median. Therefore, inferences drawn from this dichotomous age categorization may be less informative than other age categories (e.g. young adult, elderly) which were not explicitly examined. Strengths of our study include the use of the Jackson Heart Study, a large, well-characterized cohort of African Americans, which is unique in its detailed measurements of psychosocial factors related to health.

## Conclusion

Among African Americans with CKD or at increased risk of CKD, those who were younger, and males were more likely to report low use of RMC. Low trust was associated with low RMC use in all age groups. Differing barriers to engagement in RMC suggest assumptions should not be made about reasons for not seeking RMC among African Americans, as the rationale behind such behaviors are likely based on more than just self-reported race alone. Efforts to identify patients at risk of CKD incidence and progression in the settings they are most likely to receive care and efforts to address attitudes and perceptions which may hinder care represent important potential targets for future CKD prevention efforts.

## Additional file

Additional file 1: **Table S1.** Characteristics of participants included vs. those excluded from the analysis. (DOCX 19 kb)

## Abbreviations

BMI: Body mass index
CKD: Chronic kidney disease
ESRD: End stage renal disease
RMC: Routine medical care
