# Supplementary material for: Low use of routine medical care among African Americans with high CKD risk: the Jackson Heart Study
Source: BMC Nephrol. 2019 Jan 10;20:11. doi: 10.1186/s12882-018-1190-0 (PMC6327442; doi:10.1186/s12882-018-1190-0)
Supplement: Supplementary file 1 — Table S1. Characteristics of participants included vs. those excluded from the analysis. (DOCX 19 kb) [file 12882_2018_1190_MOESM1_ESM.docx]

**Supplementary Table 1: Characteristics of participants included vs. those excluded from the analysis**

| **Characteristic** | **Overall***  **N=5306** | **Excluded* n=2115** | **Included**  **n=3191** | **P Value** |
| --- | --- | --- | --- | --- |
| **Sociodemographic** |  |  |  |  |
| Age, years, mean ± SD | 55.36 ± 12.85 | 50.01 ± 13.08 | 58.9 ± 11.39 | <0.01 |
| Gender, n (%) |  |  |  | 0.21 |
| Female | 3367 (63.52) | 1318 (62.46) | 2049 (64.21) |  |
| Male | 1934 (36.48) | 792 (37.54) | 1142 (35.79) |  |
| Education, n (%) |  |  |  | <0.01 |
| ≤High school diploma | 2049 (38.65) | 660 (31.28) | 1389 (43.53) |  |
| >High school diploma | 3232 (60.97) | 1430 (67.77) | 1802 (56.47) |  |
| Income class category, n (%) |  |  |  | <0.01 |
| Lower/Lower-middle | 1798 (33.92) | 659 (31.23) | 1139 (35.69) |  |
| Upper-middle/Upper | 2683 (50.61) | 1135 (53.79) | 1548 (48.51) |  |
| **Comorbidities & Behaviors** |  |  |  |  |
| BMI, kg/m^2^, mean ± SD | 31.75 ± 7.24 | 30.5 ± 7.03 | 32.58 ± 7.26 | <0.01 |
| Tobacco use, n (%) |  |  |  | <0.01 |
| Never | 3574 (67.42) | 1498 (71) | 2076 (65.06) |  |
| Former | 1023 (19.3) | 328 (15.55) | 695 (21.78) |  |
| Current | 693 (13.07) | 273 (12.94) | 420 (13.16) |  |
| Hypertension, n (%) | 3188 (60.14) | 256 (12.13) | 2932 (91.88) | <0.01 |
| Diabetes, n (%) | 1152 (21.73) | 83 (3.93) | 1069 (33.5) | <0.01 |
| CVD, n (%) | 572 (10.79) | 122 (5.78) | 450 (14.1) | <0.01 |
| CKD, n (%) | 663 (12.51) | 72 (3.41) | 591 (18.52) | <0.01 |
| CKD awareness, n (%) | 271 (5.11) | 99 (4.69) | 172 (5.39) | 0.48 |
| **Health care access and utilization** |  |  |  |  |
| Health insurance status, n (%) |  |  |  | <0.01 |
| No | 705 (13.3) | 320 (15.17) | 385 (12.07) |  |
| Yes | 4571 (86.23) | 1765 (83.65) | 2806 (87.93) |  |
| Insurance type, n (%) |  |  |  |  |
| Medicare | 1465 (27.64) | 337 (15.97) | 1128 (35.35) | <0.01 |
| Medicaid | 636 (12) | 157 (7.44) | 479 (15.01) | <0.01 |
| Private | 3564 (67.23) | 1512 (71.66) | 2052 (64.31) | <0.01 |
| **Psychosocial factors** |  |  |  |  |
| Stress, mean ± SD | 5.14 ± 4.38 | 5.49 ± 4.44 | 4.91 ± 4.34 | <0.01 |
| Anger, mean ± SD | 1.64 ± 0.66 | 1.63 ± 0.65 | 1.65 ± 0.67 | 0.4 |
| Discrimination, mean ± SD |  |  |  |  |
| Daily | 9.69 ± 9.14 | 10.19 ± 9.15 | 9.38 ± 9.12 | <0.01 |
| Lifetime | 2.94 ± 2.12 | 2.88 ± 2.15 | 2.97 ± 2.1 | 0.13 |
| Burden of lifetime | 3.74 ± 2.44 | 3.63 ± 2.34 | 3.81 ± 2.49 | 0.01 |
| *Does not include missing data  BMI, body mass index; CVD, cardiovascular disease; CKD, chronic kidney disease | | | | |
